# Supplementary material for: Low frequency variants associated with leukocyte telomere length in the Singapore Chinese population
Source: Commun Biol. 2021 May 3;4:519. doi: 10.1038/s42003-021-02056-7 (PMC8093266; doi:10.1038/s42003-021-02056-7)

**Supplementary table 1:** Demographic information of study datasets. Data presented and mean (SD).						
	SCHS Discovery	SCHS Replication	SCHS CAD cases	SCHS CAD controls	SMART2D	DN
	N = 20,177	N = 1,840	N = 704	N = 1,224	N = 969	N = 619
Ethnicity	Singaporean Chinese					
Age (years)	55.06 (7.45)	54.99 (7.52)	59.54 (7.92)	59.05 (7.82)	58.68 (11.51)	58.24 (12.25)
Male (%)	44.57	37.94	64.68	62.95	54.75	60.57
Smoking						
ever (%)	10.92	9.54	14.04	15.80	-	-
current (%)	17.50	14.69	33.76	22.96		
LTL	1.02 (0.22)	1.05 (0.26)	1.00 (0.23)	1.02 (0.24)	0.71 (0.31)	0.85 (0.32)

LTL: leucocyte telomere length

Supplementary table 2: Sample QC procedures for the SCHS study samples.

	SCHS Discovery	SCHS Replication	SCHS CAD
Total samples genotyped	25,273	2,035	2,136
Samples with call-rate < 95%	176	26	10
Samples with exteremes in heterozygosity (>3SD or <-3SD)	236	22	20
Cryptic related samples	2,902	26	67
PCA outliers	287	33	36
Samples pass QC	21,672	1,928	2,003
Samples with LTL data	20,231	1,844	1,933
Samples with extremes in LTL values (>3SD or <-3SD)	54	4	5
Remaining samples	20,177	1,840	1,928 (704 cases and 1,224 controls)

QC: quality control; SD: standard deviation.

Supplementary table 3: SNP QC procedures in SCHS datasets.

	SCHS Discovery	SCHS Replication	SCHS CAD
Total genotyped and imputed SNPs	133,399,947	133,414,545	132,638,542
Imputed SNPs with info score < 0.8	97,180,890	115,201,908	115,885,324
Imputed SNPs with HWE p-value < 10^{-6}	1,886	1,382	1,399
Low frequency SNPs that passed QC (MAF between 0.05% -1%)	4,598,599	4,756,872	4,396,532
Genomic inflation factor (λ)	1.0098	1.0047	1.0025

SNP: single nucleotide polymorphism; QC: quality control; HWE: Hardy-Weinberg equilibrium.

Supplementary table 4: LTL asssociated SNPs included in the weighted genetic risk score (wGRS).

	Chromosome	Position	Gene	Effect size	Variance explained %	Source
rs3219104	1	226562621	*PARP1*	0.074	0.330	Dorajoo R et al. 2019
rs11890390	2	54485682	*ACYP2*	0.040	0.130	Dorajoo R et al. 2019
rs55749605	3	101232093	*SENP7*	0.037	0.010	Li C et al. 2020
rs2293607	3	169482335	*TERC*	0.120	0.830	Dorajoo R et al. 2019
rs13137667	4	71774347	*MOB1B*	0.077	0.030	Li C et al. 2020
rs10857352	4	164101482	*NAF1*	0.064	0.150	Dorajoo R et al. 2019
rs7705526	5	1285974	*TERT*	0.118	0.800	Dorajoo R et al. 2019
rs2736176	6	31587561	*PRRC2A*	0.035	0.020	Li C et al. 2020
rs79314063	7	124481168	*POT1*	0.320	0.170	Current Study
rs7776744	7	124599749	*POT1*	0.058	0.170	Dorajoo R et al. 2019
rs79617270	8	73978144	*TERF1*	0.376	0.230	Current Study
rs7095953	10	101274425	*NKX2-3*	0.047	0.210	Dorajoo R et al. 2019
rs139620151	10	105593428	*STN1*	0.394	0.130	Current Study
rs227080	11	108247888	*ATM*	0.060	0.070	Dorajoo R et al. 2019
rs41293836	14	24721327	*TINF2*	0.233	0.840	Dorajoo R et al. 2019
rs2302588	14	73404752	*DCAF4*	0.042	0.030	Dorajoo R et al. 2019
rs3785074	16	69406986	*TERF2*	0.035	0.050	Li C et al. 2020
rs2967374	16	82209861	*MPHOSPH6*	0.056	0.100	Dorajoo R et al. 2019
rs1001761	18	662103	*TYMS*	0.042	0.160	Dorajoo R et al. 2019
rs7253490	19	22293706	*ZNF257*	0.036	0.110	Dorajoo R et al. 2019
rs41309367	20	62309554	*RTEL1*	0.058	0.150	Dorajoo R et al. 2019

Supplementary table 5: Association between rs34398311 in *POT1* gene locus and LTL, with and without adjusting for previously reported index SNP rs77767444.

	without adjustment for rs77767444			with adjustment for rs77767444		
	beta	se	P	beta	se	P
rs34398311	0.135	0.018	3.23×10^{-13}	0.084	0.018	5.63×10^{-6}

Supplementary table 6: SNP based heritability of genome-wide SNPs, common SNPs and low-frequency SNPs among 2,863 1st degree related subjects from the SCHS study

**Variants**	**SNP-based heritibility**
Common SNPs (MAF > 1%)	0.187
Low frequency SNPs (MAF between 0.05% and 1%)	0.029
Genome-wide SNPs (MAF > 0.05%)	0.206

Supplementary table 7: Association between rs186524197/rs79314063 in *POT1* gene locus and LTL, before and after conditional probability analysis.

	Original association			Conditional probability analysis		
	beta	se	P	beta	se	P
rs186524197	-0.360	0.059	1.30×10^{-9}	-0.154	0.273	0.572
rs79314063	-0.345	0.057	1.12×10^{-9}	-0.201	0.260	0.440

In conditional probability analysis, rs79314063 was included in the associaton between LTL and rs186524197 as covariate and vice versa.

Supplementary table 8: Annotation of low frequency variants identified in the study.

rsID	chromosome	position	nearest Gene	distance	function	CADD	SIFT score	SIFT prediction	Polyphen2 HDIV score	Polyphen2 HDIV prediction	LRT score	LRT prediction	MutationTaster score	MutationTaster prediction	MutationAssessor score	MutationAssessor prediction
rs79314063	7	124481168	*POT1*	0	exonic	22.5	0.014	Deleteriou	0.716	Possibly damaging	0.114	Neutral	0.882	Disease causing	2.14	Predicted functional (M, medium)
rs186524197	7	124431653	*GPR37*	25971	intergenic	0.05	NA	NA	NA	NA	NA	NA	NA	NA	NA	NA
rs79617270	8	73978144	*SBSPON*	0	UTR3	10.45	NA	NA	NA	NA	NA	NA	NA	NA	NA	NA
rs139620151	10	105593428	*SH3PXD2A*	0	intronic	3.286	NA	NA	NA	NA	NA	NA	NA	NA	NA	NA

CADD: Combined Annotation Dependent Depletion.

Supplementary table 9: Solvent accessibility score of amino acids annotated in the OB-fold, described to stack the ssDNA (T1-G10) and binding to TPP1 for the wild type (wt) POT1 protein and for the putative POT1 proteins p.(D410H). Changes from exposed to buried sites (and vice versa) are highlighted in yellow.

	Position	Widetype	p.(D410H)
	146	Exposed	Exposed
	147	Exposed	Exposed
	148	Exposed	Exposed
OB fold	149	Exposed	Exposed
	150	Buried	Buried
	151	Exposed	Exposed
	152	Exposed	Exposed
T1; T2	62	Buried	Buried
A3; G4	89	Buried	Buried
G5; G6	31	Buried	Buried
	271	Exposed	Exposed
T7	161	Buried	Buried
	245	Exposed	Exposed
T8; A9	266	Buried	Buried
G10	223	Buried	Buried
	330	Exposed	Exposed
	331	Exposed	Exposed
	332	Exposed	Exposed
	333	Exposed	Exposed
	334	Buried	Buried
	335	Exposed	Exposed
	336	Exposed	Buried
	337	Exposed	Exposed
	338	Buried	Buried
	339	Buried	Buried
	340	Exposed	Exposed
	341	Exposed	Exposed
	342	Exposed	Exposed
	343	Buried	Buried
	344	Exposed	Exposed
	345	Exposed	Exposed
	346	Exposed	Exposed
	347	Exposed	Exposed
	348	Buried	Buried
	349	Exposed	Exposed
	350	Exposed	Exposed
	351	Buried	Buried
	352	Exposed	Exposed
	353	Exposed	Exposed
	354	Exposed	Exposed
	355	Exposed	Exposed
	356	Exposed	Exposed
	357	Exposed	Exposed
	358	Exposed	Exposed

		359	Exposed	Exposed
		360	Buried	Buried
		361	Exposed	Exposed
		362	Buried	Buried
		363	Exposed	Exposed
		364	Buried	Buried
		365	Exposed	Exposed
		366	Buried	Buried
		367	Exposed	Exposed
		368	Exposed	Exposed
		369	Exposed	Exposed
		370	Exposed	Exposed
		371	Buried	Buried
		372	Exposed	Exposed
		373	Exposed	Exposed
		374	Buried	Buried
		375	Exposed	Exposed
		376	Exposed	Exposed
		377	Buried	Buried
		378	Buried	Buried
		379	Exposed	Exposed
		380	Exposed	Exposed
		381	Buried	Buried
		382	Buried	Buried
		383	Exposed	Exposed
		384	Exposed	Exposed
		385	Buried	Buried
		386	Exposed	Exposed
		387	Exposed	Exposed
		388	Buried	Buried
		389	Exposed	Exposed
		390	Exposed	Exposed
		391	Buried	Exposed
		392	Exposed	Exposed
		393	Exposed	Exposed
		394	Exposed	Exposed
		395	Exposed	Exposed
		396	Buried	Exposed
		397	Buried	Buried
		398	Exposed	Exposed
		399	Exposed	Buried
		400	Buried	Buried
		401	Buried	Buried
		402	Exposed	Exposed
		403	Exposed	Exposed
		404	Buried	Buried
		405	Buried	Buried
		406	Exposed	Exposed
		407	Exposed	Exposed
		408	Exposed	Exposed

		409	Exposed	Exposed
		410	Exposed	Exposed
		411	Exposed	Exposed
		412	Exposed	Exposed
		413	Buried	Exposed
		414	Exposed	Exposed
		415	Exposed	Exposed
		416	Buried	Buried
		417	Buried	Exposed
		418	Exposed	Buried
		419	Buried	Exposed
		420	Exposed	Exposed
		421	Exposed	Exposed
		422	Exposed	Exposed
		423	Exposed	Exposed
		424	Exposed	Exposed
		425	Exposed	Exposed
		426	Exposed	Exposed
		427	Exposed	Exposed
		428	Exposed	Exposed
		429	Exposed	Exposed
		430	Exposed	Exposed
		431	Exposed	Exposed
		432	Exposed	Exposed
		433	Exposed	Exposed
		434	Buried	Buried
		435	Buried	Buried
		436	Buried	Buried
		437	Exposed	Exposed
		438	Buried	Buried
		439	Buried	Buried
		440	Exposed	Buried
		441	Exposed	Exposed
		442	Exposed	Exposed
		443	Exposed	Exposed
		444	Buried	Buried
		445	Exposed	Exposed
		446	Exposed	Exposed
		447	Exposed	Exposed
		448	Exposed	Exposed
		449	Exposed	Exposed
		450	Exposed	Exposed
		451	Buried	Exposed
		452	Buried	Buried
		453	Buried	Buried
		454	Buried	Buried
		455	Buried	Buried
		456	Buried	Buried
		457	Buried	Buried
		458	Buried	Buried

TPP1 binding	459	Buried	Buried
	460	Buried	Buried
	461	Exposed	Exposed
	462	Exposed	Exposed
	463	Buried	Buried
	464	Buried	Buried
	465	Exposed	Exposed
	466	Buried	Buried
	467	Buried	Buried
	468	Exposed	Exposed
	469	Exposed	Exposed
	470	Buried	Buried
	471	Exposed	Exposed
	472	Exposed	Exposed
	473	Buried	Buried
	474	Buried	Buried
	475	Buried	Buried
	476	Buried	Buried
	477	Exposed	Exposed
	478	Exposed	Exposed
	479	Exposed	Exposed
	480	Exposed	Exposed
	481	Exposed	Exposed
	482	Exposed	Exposed
	483	Buried	Buried
	484	Exposed	Exposed
	485	Buried	Buried
	486	Buried	Buried
	487	Exposed	Exposed
	488	Buried	Buried
	489	Buried	Buried
	490	Buried	Buried
	491	Buried	Buried
	492	Buried	Buried
	493	Buried	Buried
	494	Buried	Buried
	495	Exposed	Exposed
	496	Buried	Exposed
	497	Exposed	Exposed
	498	Buried	Buried
	499	Exposed	Exposed
	500	Exposed	Exposed
	501	Buried	Buried
	502	Buried	Buried
	503	Buried	Buried
	504	Exposed	Exposed
	505	Exposed	Exposed
	506	Buried	Buried
	507	Exposed	Exposed
	508	Exposed	Exposed

		509	Exposed	Exposed
		510	Exposed	Exposed
		511	Exposed	Exposed
		512	Buried	Buried
		513	Exposed	Exposed
		514	Exposed	Exposed
		515	Buried	Buried
		516	Exposed	Exposed
		517	Exposed	Exposed
		518	Buried	Buried
		519	Exposed	Exposed
		520	Exposed	Exposed
		521	Exposed	Exposed
		522	Exposed	Exposed
		523	Exposed	Exposed
		524	Exposed	Exposed
		525	Exposed	Exposed
		526	Exposed	Exposed
		527	Exposed	Exposed
		528	Exposed	Exposed
		529	Buried	Buried
		530	Buried	Buried
		531	Exposed	Exposed
		532	Exposed	Exposed
		533	Exposed	Exposed
		534	Buried	Exposed
		535	Buried	Buried
		536	Exposed	Exposed
		537	Exposed	Exposed
		538	Buried	Buried
		539	Exposed	Exposed
		540	Buried	Buried
		541	Buried	Buried
		542	Buried	Buried
		543	Buried	Buried
		544	Buried	Buried
		545	Exposed	Exposed
		546	Buried	Buried
		547	Exposed	Exposed
		548	Buried	Buried
		549	Exposed	Exposed
		550	Exposed	Exposed
		551	Buried	Buried
		552	Exposed	Exposed
		553	Exposed	Buried
		554	Exposed	Exposed
		555	Buried	Buried
		556	Exposed	Exposed
		557	Buried	Buried
		558	Buried	Buried

	559	Buried	Buried
	560	Exposed	Exposed
	561	Exposed	Exposed
	562	Buried	Buried
	563	Exposed	Exposed
	564	Exposed	Exposed
	565	Buried	Buried
	566	Buried	Buried
	567	Exposed	Exposed
	568	Buried	Buried
	569	Exposed	Exposed
	570	Buried	Buried
	571	Exposed	Exposed
	572	Exposed	Exposed
	573	Buried	Buried
	574	Buried	Buried
	575	Buried	Exposed
	576	Exposed	Exposed
	577	Exposed	Exposed
	578	Exposed	Exposed
	579	Buried	Buried
	580	Buried	Buried
	581	Exposed	Exposed
	582	Exposed	Exposed
	583	Buried	Buried
	584	Exposed	Exposed
	585	Exposed	Exposed
	586	Buried	Buried
	587	Buried	Buried
	588	Exposed	Exposed
	589	Exposed	Exposed
	590	Buried	Buried
	591	Buried	Buried
	592	Exposed	Buried
	593	Exposed	Exposed
	594	Exposed	Exposed
	595	Exposed	Exposed
	596	Exposed	Exposed
	597	Buried	Buried
	598	Exposed	Exposed
	599	Exposed	Exposed
	600	Exposed	Exposed
	601	Buried	Buried
	602	Buried	Buried
	603	Buried	Buried
	604	Exposed	Buried
	605	Buried	Buried
	606	Buried	Buried
	607	Buried	Buried
	608	Exposed	Exposed

	609	Buried	Buried
	610	Buried	Buried
	611	Exposed	Exposed
	612	Buried	Buried
	613	Exposed	Exposed
	614	Exposed	Exposed
	615	Exposed	Exposed
	616	Exposed	Exposed
	617	Exposed	Exposed
	618	Exposed	Exposed
	619	Exposed	Exposed
	620	Buried	Buried
	621	Buried	Buried
	622	Buried	Buried
	623	Exposed	Exposed
	624	Buried	Buried
	625	Buried	Buried
	626	Exposed	Exposed
	627	Buried	Buried
	628	Exposed	Buried
	629	Buried	Buried
	630	Buried	Buried
	631	Exposed	Exposed
	632	Exposed	Exposed
	633	Buried	Buried
	634	Exposed	Exposed

Supplementary table 10: Summary statistics of LTL association for low frequency variants identified within the SCHS Discovery dataset adjusted for the genotypes of the reported common index variants using conditional probability analysis.

	Rare variant genome-wide hits			Common variant genome-wide hits						
Chr	SNP	Position	MAF	SNP	Position	MAF	r^2	Beta	Se	P
7	rs79314063	124481168	0.008	rs7776744	124599749	0.422	0.065	-0.347	0.066	1.70×10^{-7}
8	rs79617270	73978144	0.008	rs28365964	73920883	0.017	0.664	0.206	0.076	0.007
10	rs139620151	105593428	0.006	rs12415148	105680586	0.058	0.248	0.183	0.077	0.017

Chr: Chromosome; MAF: Minor allele frequency.

Supplementary table 11: Summary statistics of novel low frequency SNPs and previously identified common index SNPs in the SCHS Discovery dataset.

SNP	Remarks	Chr	Position	Effect allele	EAF	beta	se	P
rs79314063	Novel low frequency SNP	7	124481168	G	0.008	-0.345	0.057	1.12×10^{-9}
rs7776744	Common SNP	7	124599749	G	0.578	-0.065	0.010	9.76×10^{-12}
rs79617270	Novel low frequency SNP	8	73978144	G	0.008	0.385	0.054	8.63×10^{-13}
rs28365964	Common SNP	8	73920883	C	0.017	0.284	0.038	3.46×10^{-14}
rs139620151	Novel low frequency SNP	10	105593428	A	0.006	0.384	0.067	1.10×10^{-8}
rs12415148	Common SNP	10	105680586	C	0.058	0.220	0.020	4.70×10^{-26}

Supplementary table 12: Significant 3D chromatin interaction mapping identified for rs79617270 and rs139620151.

region 1	region 2	FDR	type	Database	tissue/cell	SNPs	genes
8:73960001-74000000	8:72720001-72760000	5.34×10^{-12}	HiC	GSE87112	Mesenchymal_Stem_Cell	rs79617270	*MSC-AS1, MSC*
8:73960001-74000000	8:73440001-73480000	2.30×10^{-12}	HiC	GSE87112	Mesenchymal_Stem_Cell	rs79617270	*KCNB2*
8:73960001-74000000	8:73440001-73480000	7.73×10^{-9}	HiC	GSE87112	Mesendoderm	rs79617270	*KCNB2*
8:73960001-74000000	8:74000001-74040000	3.30×10^{-9}	HiC	GSE87112	Left_Ventricle	rs79617270	*SBSPON*
8:73960001-74000000	8:74000001-74040000	6.93×10^{-8}	HiC	GSE87112	Liver	rs79617270	*SBSPON*
8:73960001-74000000	8:74000001-74040000	5.34×10^{-11}	HiC	GSE87112	GM12878	rs79617270	*SBSPON*
8:73960001-74000000	8:74000001-74040000	1.77×10^{-16}	HiC	GSE87112	IMR90	rs79617270	*SBSPON*
8:73960001-74000000	8:74000001-74040000	2.46×10^{-60}	HiC	GSE87112	Mesenchymal_Stem_Cell	rs79617270	*SBSPON*
8:73960001-74000000	8:74000001-74040000	1.87×10^{-44}	HiC	GSE87112	Mesendoderm	rs79617270	*SBSPON*
8:73960001-74000000	8:74000001-74040000	1.24×10^{-28}	HiC	GSE87112	Trophoblast-like_Cell	rs79617270	*SBSPON*
8:73960001-74000000	8:74000001-74040000	1.49×10^{-97}	HiC	GSE87112	hESC	rs79617270	*SBSPON*
8:73960001-74000000	8:73920001-73960000	5.94×10^{-9}	HiC	GSE87112	GM12878	rs79617270	*TERF1*
8:73960001-74000000	8:73920001-73960000	6.50×10^{-42}	HiC	GSE87112	Mesenchymal_Stem_Cell	rs79617270	*TERF1*
8:73960001-74000000	8:73920001-73960000	1.14×10^{-70}	HiC	GSE87112	Mesendoderm	rs79617270	*TERF1*
8:73960001-74000000	8:73920001-73960000	2.78×10^{-15}	HiC	GSE87112	Trophoblast-like_Cell	rs79617270	*TERF1*
8:73960001-74000000	8:73920001-73960000	1.10×10^{-36}	HiC	GSE87112	hESC	rs79617270	*TERF1*
8:73960001-74000000	8:75200001-75240000	2.24×10^{-8}	HiC	GSE87112	IMR90	rs79617270	*JPH1, GDAP1*
8:73960001-74000000	8:72960001-73000000	8.77×10^{-10}	HiC	GSE87112	Mesenchymal_Stem_Cell	rs79617270	*TRPA1*
10:105560001-105600000	10:105640001-105680000	2.06×10^{-7}	HiC	GSE87112	IMR90	rs139620151	*STN1/OBFC1*
10:105560001-105600000	10:105600001-105640000	2.39×10^{-14}	HiC	GSE87112	IMR90	rs139620151	*SH3PXD2A*
10:105560001-105600000	10:106000001-106040000	3.99×10^{-7}	HiC	GSE87112	IMR90	rs139620151	*GSTO2*

Region 1: One end of a significant interaction overlapping with one of the candidate SNPs; FDR: false discovery rate.

Supplementary table 13: Summary statistics of association between low frequency index variants and LTL in individual dataset at replication stage.

				SCHS Replication N = 1,840				SCHS CAD cases N = 704				SCHS CAD controls N = 1,224				SMART2D N = 969				DN N = 619				Replication meta-analysis N = 5,356		
	Chromosome	Position	TA	TAF	beta	se	P	TAF	beta	se	P	TAF	beta	se	P	TAF	beta	se	P	TAF	beta	se	P	beta	se	P
rs79314063	7	124481168	G	0.008	-0.105	0.170	0.536	0.006	-0.272	0.363	0.454	0.002	-0.404	0.306	0.187	0.002	-0.194	0.487	0.691	0.002	-0.264	0.971	0.785	-0.191	0.131	0.146
rs79617270	8	73978144	G	0.009	0.325	0.168	0.052	0.006	0.030	0.390	0.938	0.0004	0.785	0.310	0.011	0.005	0.054	0.327	0.868	0.004	0.297	0.485	0.541	0.328	0.123	0.008
rs139620151	10	105593428	A	0.002	0.106	0.431	0.807	0.006	0.584	0.340	0.085	0.006	0.493	0.257	0.055	0.003	0.270	0.437	0.536	0.003	0.825	0.558	0.140	0.456	0.163	0.005

TA: test allele; TAF: test allele frequency.

Supplementary table 14: Summary statistics of association between low frequency index variants and LTL in SingHEART/Biobank.

					N = 154		
rsid	chromosome	Gene	Position	Effect allele	beta	se	*P*
rs79314063	7	*POT1*	124481168	G	-587	339.584	0.090
rs79617270	8	*TERF1 / SBSPON*	73978144	G	370	221.063	0.100
rs139620151	10	*STN1 / SH3PXD2A*	105593428	A	1239	471.185	0.009

Beta showed the base pair change of telomere length per effect allele.

Supplementary table 15: Association of identified SNPs with LTL after further adjustment for smoking status (never smokers or ex-smokers vs current).

SNP	Chr	Position	Gene	TA	TAF	SCHS Discovery			SCHS CAD			SCHS Replication			Meta-analysis			
						Beta	Se	P	Beta	Se	P	Beta	Se	P	Beta	Se	P	Q_{pval}
rs79314063	7	1.24E+08	*POT1*	G	0.007	-0.345	0.057	1.06×10^{-9}	-0.262	0.456	0.566	-0.099	0.169	0.557	-0.319	0.053	1.97×10^{-9}	0.384
rs79617270	8	73978144	*TERF1 / SBSPON*	G	0.008	0.384	0.054	9.10×10^{-13}	0.494	0.268	0.050	0.326	0.168	0.052	0.383	0.050	2.73×10^{-14}	0.865
rs139620151	10	1.06E+08	*STN1 / SH3PXD2A*	A	0.006	0.383	0.067	1.09×10^{-8}	0.486	0.257	0.058	0.110	0.431	0.799	0.384	0.064	2.23×10^{-9}	0.755

**Supplementary table 16:** Replication of previously reported LTL rare variants in *ATM* and *RPL8* gene region, first identified in European population samples								
	Chromosome	Position	Nearest Gene	Effect allele	MAF	Beta	Se	P
rs144114619	6	26408145	*BTN3A1*	Monomorphic in East Asians				
rs763648407	8	146017753	*RPL8*	A	0.006	-0.066	0.068	0.332
rs185270276	11	108263828	*C11orf65*	C	0.015	0.060	0.038	0.122
rs12365364	11	108004687	*ACAT1*	A	0.015	0.058	0.039	0.135
rs79119325	11	108032614	*NPAT*	Monomorphic in East Asians				
rs3092910	11	108180917	*ATM,C11orf65*	C	0.016	0.049	0.038	0.196
rs3218711	11	108236264	*ATM,C11orf65*	G	0.016	0.052	0.038	0.168
rs11212668	11	108352576	*KDELC2*	C	0.015	0.071	0.039	0.066
rs12146512	11	108384666	*EXPH5*	Monomorphic in East Asians				
rs2234993	11	108129599	*ATM*	G	0.015	0.051	0.038	0.176
rs3218670	11	108186743	*ATM*	A	0.0009	-0.496	0.167	0.003

Supplementary table 17: Top ten signals in group-wise gene burden tests (CMC and Zeggini gene burden tests).

		CMC				Zeggini			
		Original association		Exclude lead SNP in the region from analysis		Original association		Exclude lead SNP in the region from analysis	
Gene	Range	P	P_{adj}	P	P_{adj}	P	P_{adj}	P	P_{adj}
POT1	7:124462439-124570037,7:124462439-124570037,7:124462439-124570037,7:124462439-124570037,7:124462439-124570037	1.82×10^{-6}	0.029	0.434	1.000	4.23×10^{-8}	6.73×10^{-4}	0.403	1.000
KIF12	9:116853917-116861337	1.26×10^{-4}	1.000	-	-	1.26×10^{-4}	1.000	-	-
KIAA0825	5:93854917-93954309,5:93486555-93954309	1.42×10^{-4}	1.000	-	-	1.42×10^{-4}	1.000	-	-
RBM25	14:73525220-73588076	3.18×10^{-4}	1.000	-	-	3.18×10^{-4}	1.000	-	-
ZNF518B	4:10441503-10459032	3.61×10^{-4}	1.000	-	-	3.32×10^{-4}	1.000	-	-
PSMD6	3:63996230-64009120	3.74×10^{-4}	1.000	-	-	3.74×10^{-4}	1.000	-	-
P4HA2	5:131528303-131563556,5:131528303-131562935,5:131528303-131563556,5:131528303-131563556,5:131528303-131562935	4.03×10^{-4}	1.000	-	-	4.03×10^{-4}	1.000	-	-
UNC119	17:26873724-26879646,17:26873724-26879646	4.53×10^{-4}	1.000	-	-	4.53×10^{-4}	1.000	-	-
LOC100130522	18:77905806-77929616,18:77905806-77936315	4.83×10^{-4}	1.000	-	-	4.83×10^{-4}	1.000	-	-
PKP4	2:159313475-159537940,2:159313475-159537940	6.72×10^{-4}	1.000	-	-	-	-	-	-
PGLYRP4	1:153302596-153321022	-	-	-	-	2.96×10^{-4}	1.000	-	-

Gene region showed level of significance after adjusting for multiple comparison was further analyzed by excluding the lead SNP in the region.

Supplementary table 18: Association of LTL associated low frequency variants with coronary artery disease in the SCHS CAD dataset.

		rs79314063				rs79617270				rs139620151			
	Case/Non-Case	Test allele	HR (95% CI)	P	P_{adj}	Test allele	HR (95% CI)	P	P_{adj}	Test allele	HR (95% CI)	P	P_{adj}
CAD	711 / 1,246	G	1.835 (0.820, 4.104)	0.140	0.420	G	0.844 (0.350, 2.036)	0.844	1.000	A	1.098 (0.546, 2.206)	0.793	1.000

P_{adj}: score test P adjusted for 3 tests; HR: hazard ratio; CI: confidence interval.

Supplementary table 19: Effect of SNP and wGRS on incident cancer mediated through LTL in SCHS.

	Direct effect			Indirect effect			Total effect			Proportion mediated through LTL
	Beta	Se	P	Beta	Se	P	Beta	Se	P	
Colon cancer										
rs79617270	0.921	0.288	0.001	0.046	0.022	0.041	0.967	0.287	0.001	4.76%
Lung cancer										
wGRS	0.071	0.026	0.007	0.012	0.006	0.038	0.083	0.025	0.001	14.40%
Lung adenocarcinoma										
wGRS	0.095	0.036	0.009	0.031	0.008	1.18×10^{-4}	0.126	0.035	3.60×10^{-4}	24.92%
Lung cancer										
rs7705526	0.267	0.069	9.74×10^{-5}	0.015	0.006	0.015	0.283	0.068	3.62×10^{-5}	5.47%

wGRS: weighted genetic risk score; LTL: leukocyte telomere length.

Supplementary table 20: Association of individual LTL SNPs with major incident cancers in the SCHS.

					Colon cancer			Rectal cancer			Lung cancer			Breast cancer		
					405 case/21531 non-case			249 case /21531 non-case			496 case /21531 non-case			335 case /12112 non-case		
	Chromosome	Position	Gene	Effect size	HR (95% CI)	P	P_{adj}	HR (95% CI)	P	P_{adj}	HR (95% CI)	P	P_{adj}	HR (95% CI)	P	P_{adj}
rs3219104	1	226562621	*PARP1*	0.074	1.010 (0.880, 1.160)	0.882	1.000	1.030 (0.864, 1.228)	0.740	1.000	0.947 (0.837, 1.072)	0.390	1.000	0.984 (0.845, 1.147)	0.840	1.000
rs11890390	2	54485682	*ACYP2*	0.040	0.954 (0.794, 1.145)	0.611	1.000	1.005 (0.799, 1.262)	0.968	1.000	0.992 (0.843, 1.166)	0.920	1.000	0.902 (0.735, 1.107)	0.324	1.000
rs55749605	3	101232093	*SENP7*	0.037	1.016 (0.881, 1.170)	0.831	1.000	0.986 (0.823, 1.183)	0.883	1.000	0.902 (0.792, 1.028)	0.121	1.000	1.121 (0.961, 1.308)	0.147	1.000
rs13137667	4	71774347	*MOB1B*	0.077	1.026 (0.713, 1.477)	0.888	1.000	0.862 (0.562, 1.320)	0.494	1.000	1.089 (0.778, 1.525)	0.620	1.000	0.925 (0.629, 1.359)	0.690	1.000
rs2293607	3	169482335	*TERC*	0.120	0.985 (0.857, 1.131)	0.828	1.000	1.072 (0.898, 1.279)	0.444	1.000	1.227 (1.082, 1.391)	0.001	0.101	1.074 (0.923, 1.249)	0.357	1.000
rs10857352	4	164101482	*NAF1*	0.064	1.033 (0.881, 1.210)	0.691	1.000	0.916 (0.753, 1.115)	0.383	1.000	1.113 (0.961, 1.288)	0.153	1.000	1.227 (1.023, 1.472)	0.028	1.000
rs7705526	5	1285974	*TERT*	0.118	1.086 (0.945, 1.249)	0.245	1.000	1.016 (0.850, 1.215)	0.859	1.000	1.273 (1.124, 1.442)	1.40×10^{-4}	0.010	1.202 (1.033, 1.399)	0.017	1.000
rs2736176	6	31587561	*PRRC2A*	0.035	1.072 (0.926, 1.241)	0.350	1.000	1.121 (0.931, 1.349)	0.227	1.000	1.009 (0.883, 1.153)	0.898	1.000	0.930 (0.787, 1.098)	0.389	1.000
rs7776744	7	124599749	*POT1*	0.058	1.078 (0.939, 1.239)	0.285	1.000	1.021 (0.855, 1.219)	0.819	1.000	1.004 (0.885, 1.138)	0.957	1.000	0.892 (0.764, 1.042)	0.150	1.000
rs7095953	10	101274425	*NKX2-3*	0.047	1.019 (0.886, 1.173)	0.788	1.000	0.879 (0.736, 1.049)	0.154	1.000	1.042 (0.918, 1.182)	0.528	1.000	0.819 (0.703, 0.954)	0.010	0.755
rs227080	11	108247888	*ATM*	0.060	1.106 (0.959, 1.276)	0.166	1.000	1.033 (0.862, 1.237)	0.727	1.000	0.995 (0.877, 1.129)	0.936	1.000	1.065 (0.911, 1.245)	0.429	1.000
rs41293836	14	24721327	*TINF2*	0.233	1.034 (0.802, 1.332)	0.799	1.000	1.043 (0.755, 1.443)	0.797	1.000	1.034 (0.821, 1.302)	0.778	1.000	1.043 (0.792, 1.373)	0.763	1.000
rs2302588	14	73404752	*DCAF4*	0.042	1.014 (0.857, 1.200)	0.873	1.000	0.754 (0.595, 0.957)	0.020	1.000	1.020 (0.875, 1.188)	0.801	1.000	1.113 (0.929, 1.332)	0.246	1.000
rs3785074	16	69406986	*TERF2*	0.035	1.013 (0.794, 1.291)	0.919	1.000	0.957 (0.695, 1.316)	0.785	1.000	0.939 (0.749, 1.179)	0.589	1.000	0.933 (0.707, 1.232)	0.625	1.000
rs2967374	16	82209861	*MPHOSPH6*	0.056	1.064 (0.883, 1.284)	0.513	1.000	0.954 (0.744, 1.222)	0.707	1.000	0.924 (0.774, 1.104)	0.384	1.000	1.004 (0.814, 1.238)	0.972	1.000
rs1001761	18	662103	*TYMS*	0.042	1.026 (0.881, 1.195)	0.738	1.000	1.029 (0.847, 1.250)	0.777	1.000	0.870 (0.754, 1.005)	0.058	1.000	0.838 (0.705, 0.997)	0.046	1.000
rs7253490	19	22293706	*ZNF257*	0.036	1.076 (0.932, 1.243)	0.318	1.000	0.928 (0.769, 1.120)	0.435	1.000	1.057 (0.928, 1.204)	0.406	1.000	0.990 (0.843, 1.164)	0.907	1.000
rs41309367	20	62309554	*RTEL1*	0.058	1.080 (0.922, 1.266)	0.338	1.000	0.897 (0.739, 1.088)	0.268	1.000	0.997 (0.866, 1.147)	0.963	1.000	0.948 (0.801, 1.124)	0.541	1.000

P_{adj}: score test P adjusted for 72 tests; HR: hazard ratio; CI: confidence interval.

Supplementary Figure 1. Correlation of imputed common biallelic SNPs (MAF > 1%) frequencies between the two combined reference panels (the cosmopolitan 1000 Genomes reference panels, Phase 3 and local Singapore population specific reference panels) and 1000 Genomes East-Asian reference populations.

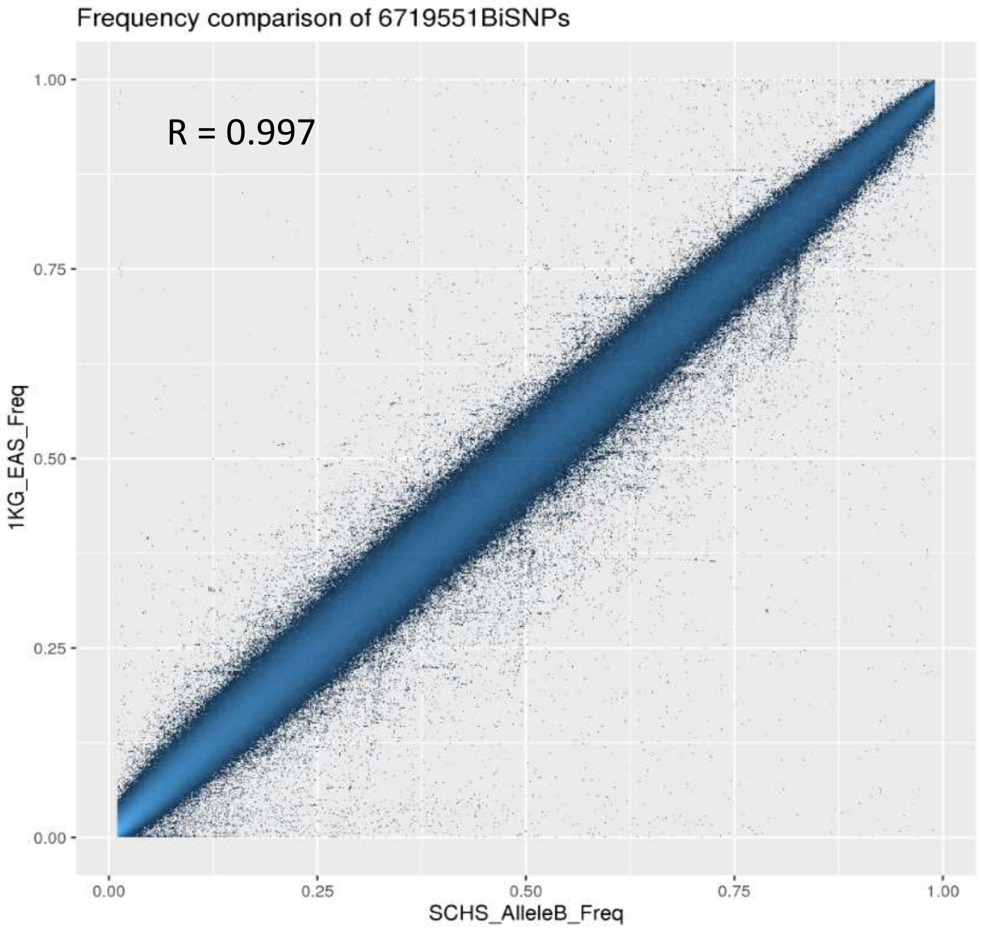

Supplementary Figure 2. LTL associations for common variants utilizing the two panel imputation procedure in the SCHS discovery dataset. **a.** All 10 genome-wide hits previously reported were recapitulated [1]. **b.** QQ-plot of observed compared

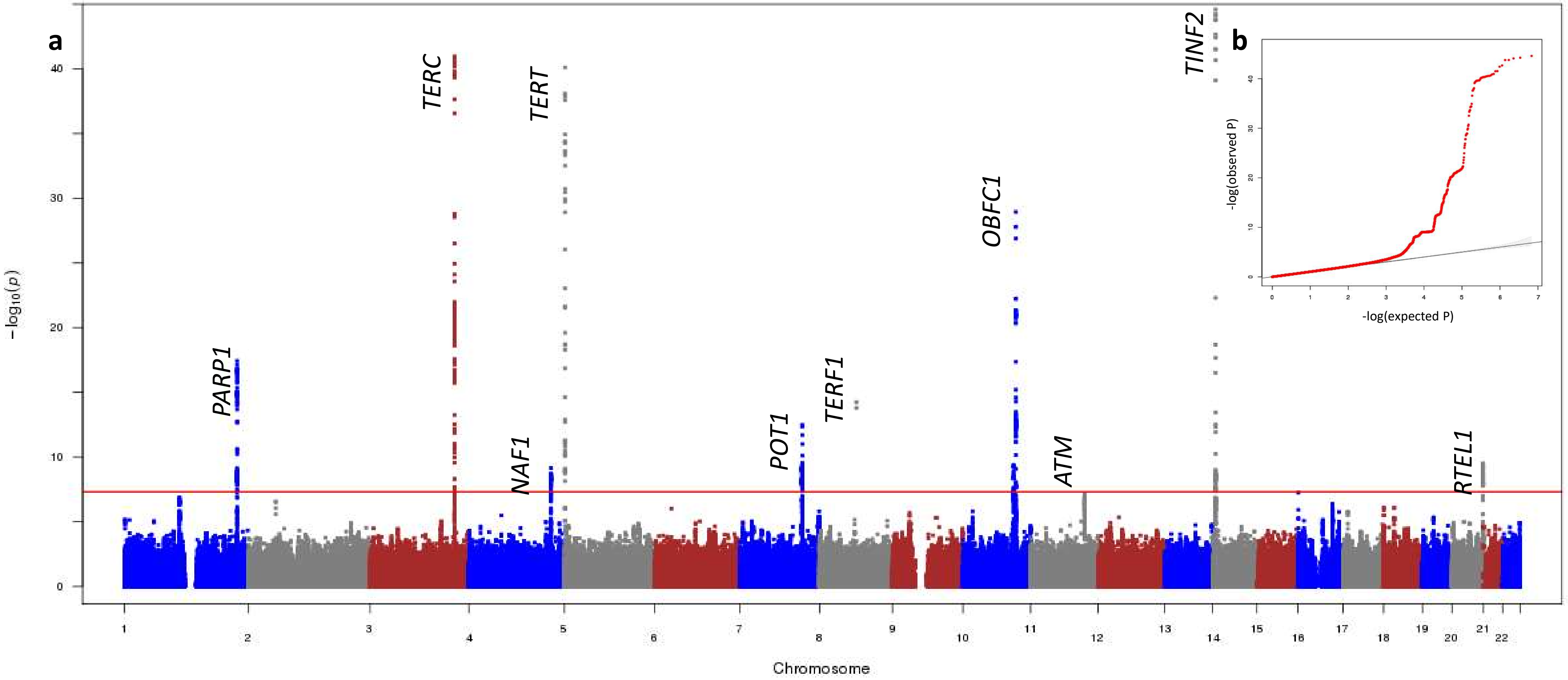

1. Dorajoo, R., X. Chang, R.L. Gurung, et al., *Loci for human leukocyte telomere length in the Singaporean Chinese population and trans-ethnic genetic studies.* Nature communications, 2019. **10**(1): p. 2491 DOI: 10.1038/s41467-019-10443-2.

Supplementary Figure 3. Regional common SNP associations at loci reported the same index SNP [1]. **a.** *TERT* (rs7705526, chr5) **b.** *TINF2* (rs41293836, chr14) and **c.** *RTEL1* (rs41309367, chr20). Plots plotted using LocusZoom (http://csg.sph.umich.edu/locuszoom/).

a

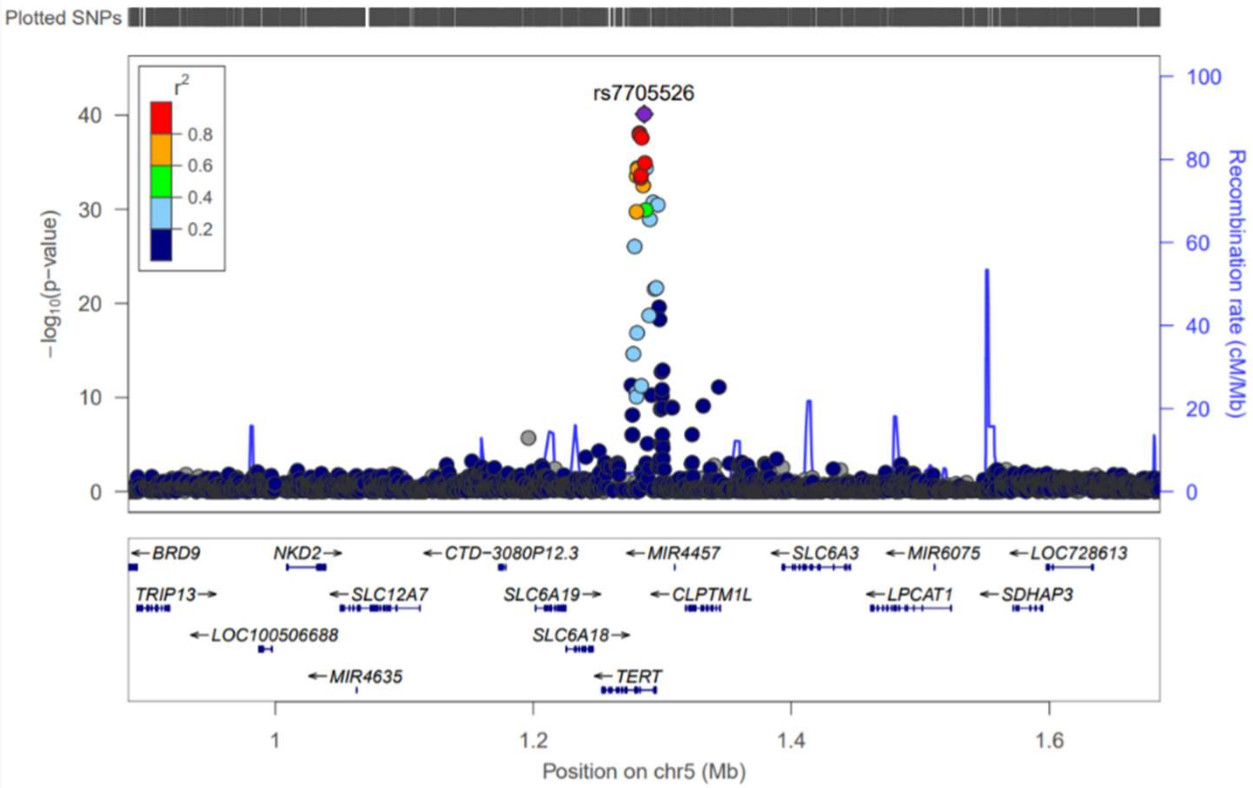

1. Dorajoo, R., X. Chang, R.L. Gurung, et al., *Loci for human leukocyte telomere length in the Singaporean Chinese population and trans-ethnic genetic studies.* Nature communications, 2019. **10**(1): p. 2491 DOI: 10.1038/s41467-019-10443-2.

Supplementary Figure 3. Regional common SNP associations at loci reported the same index SNP [1]. **a.** *TERT* (rs7705526, chr5) **b.** *TINF2* (rs41293836, chr14) and **c.** *RTEL1* (rs41309367, chr20). Plots plotted using LocusZoom (http://csg.sph.umich.edu/locuszoom/).

b

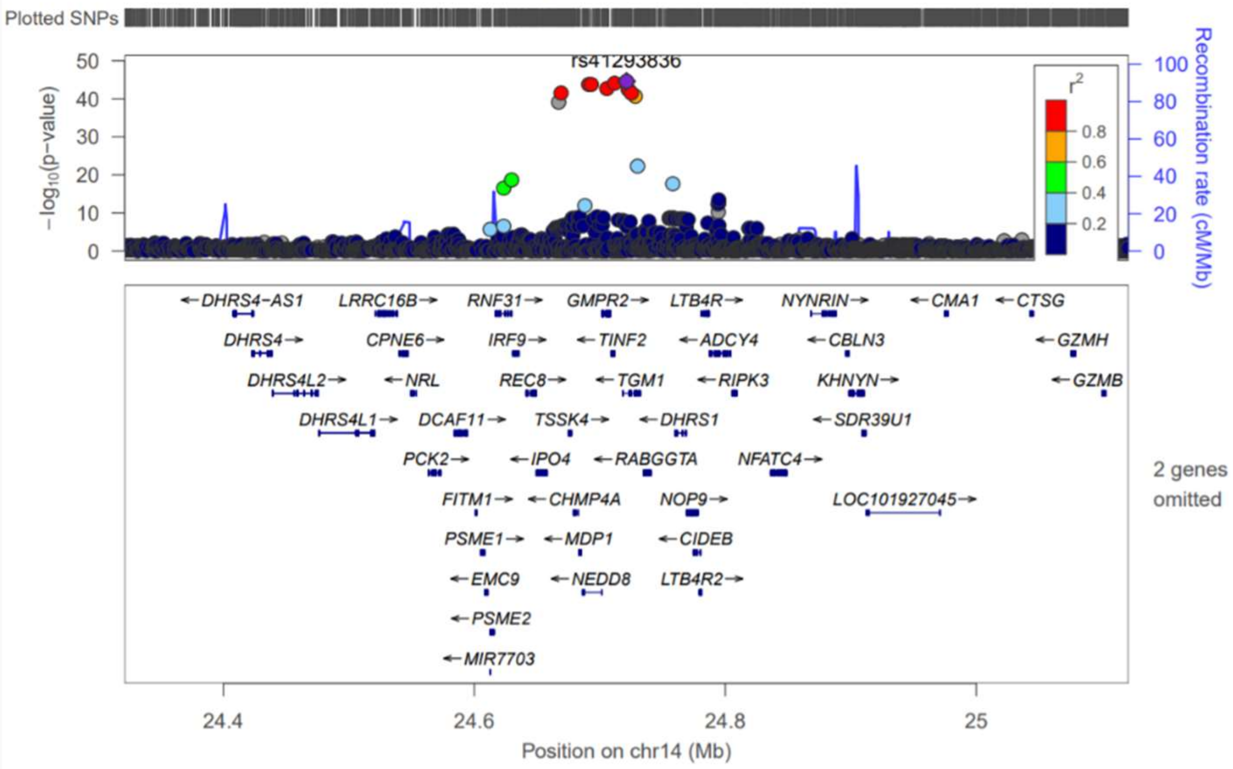

1. Dorajoo, R., X. Chang, R.L. Gurung, et al., *Loci for human leukocyte telomere length in the Singaporean Chinese population and trans-ethnic genetic studies.* Nature communications, 2019. **10**(1): p. 2491 DOI: 10.1038/s41467-019-10443-2.

Supplementary Figure 3. Regional common SNP associations at loci reported the same index SNP [1]. **a.** *TERT* (rs7705526, chr5) **b.** *TINF2* (rs41293836, chr14) and **c.** *RTEL1* (rs41309367, chr20). Plots plotted using LocusZoom (http://csg.sph.umich.edu/locuszoom/).

c

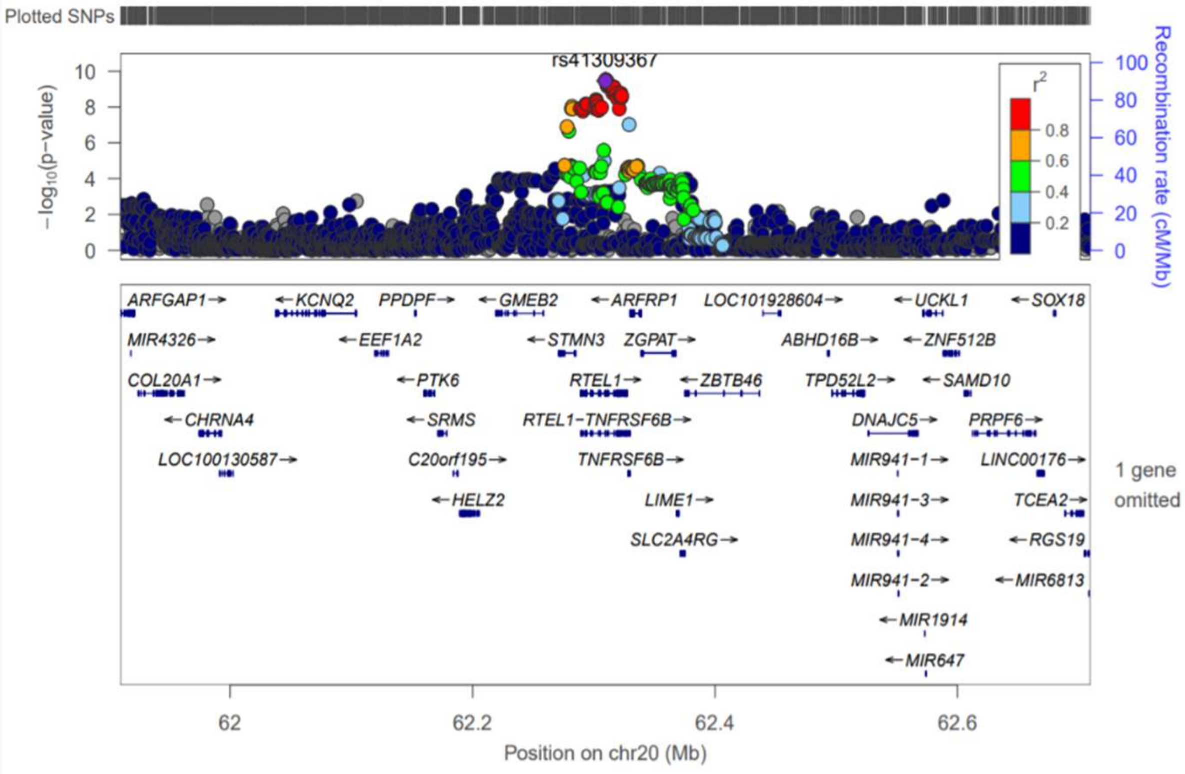

1. Dorajoo, R., X. Chang, R.L. Gurung, et al., *Loci for human leukocyte telomere length in the Singaporean Chinese population and trans-ethnic genetic studies.* Nature communications, 2019. **10**(1): p. 2491 DOI: 10.1038/s41467-019-10443-2.

Supplementary Figure 4. Regional common SNP associations at loci identified new common SNPs in linkage disequilibrium with previous reported index SNPs [1]. **a.** *PARP1* (rs3219104, chr1) **b.** *TERC* (rs2293607, chr3) **c.** *NAF1* (rs10857352, chr4) **d.** *TERF1* (rs28365964, chr8) **e.** *OBFC1* (rs12415148, chr10) and **f.** *ATM* (rs227080, chr11). Plots plotted using LocusZoom (http://csg.sph.umich.edu/locuszoom/). Previous index SNPs were indicated as purple diamond. New index SNPs were circled and indicated in green.

a

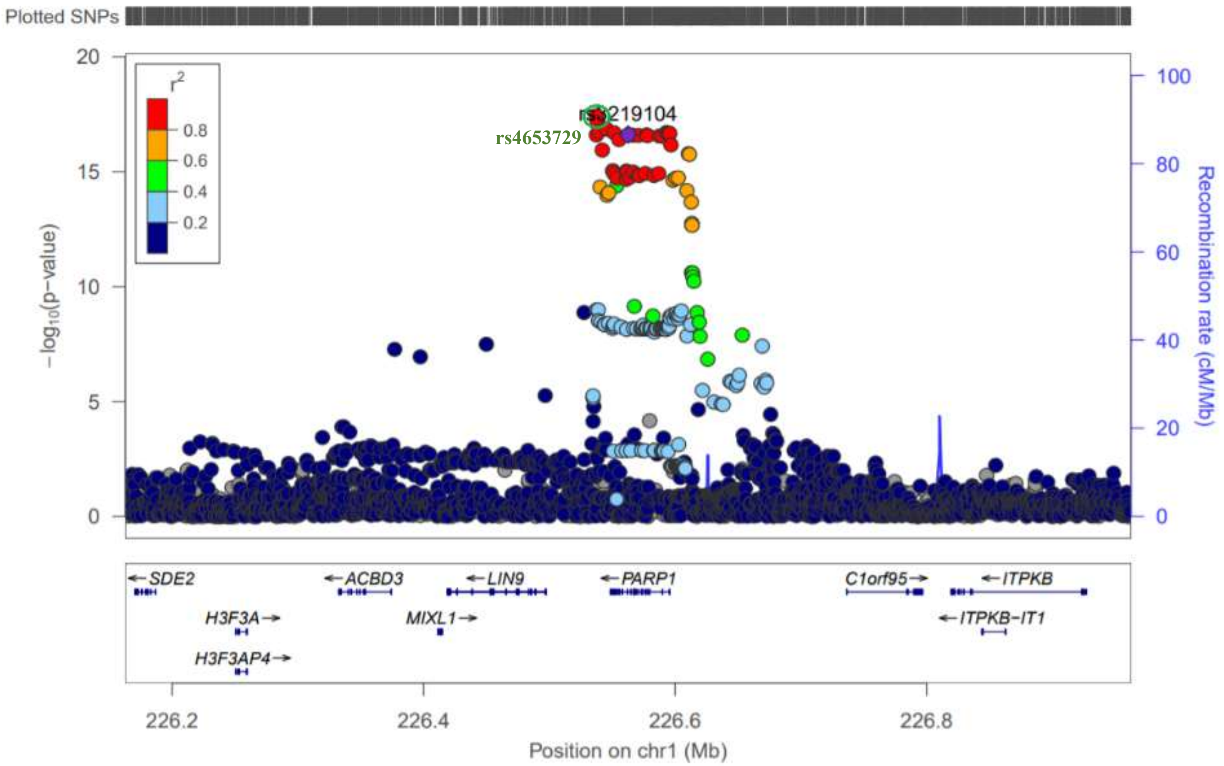

1. Dorajoo, R., X. Chang, R.L. Gurung, et al., *Loci for human leukocyte telomere length in the Singaporean Chinese population and trans-ethnic genetic studies.* Nature communications, 2019. **10**(1): p. 2491 DOI: 10.1038/s41467-019-10443-2.

Supplementary Figure 4. Regional common SNP associations at loci identified new common SNPs in linkage disequilibrium with previous reported index SNPs [1]. **a.** *PARP1* (rs3219104, chr1) **b.** *TERC* (rs2293607, chr3) **c.** *NAF1* (rs10857352, chr4) **d.** *TERF1* (rs28365964, chr8) **e.** *OBFC1* (rs12415148, chr10) and **f.** *ATM* (rs227080, chr11). Plots plotted using LocusZoom (http://csg.sph.umich.edu/locuszoom/). Previous index SNPs were indicated as purple diamond. New index SNPs were circled and indicated in green.

b

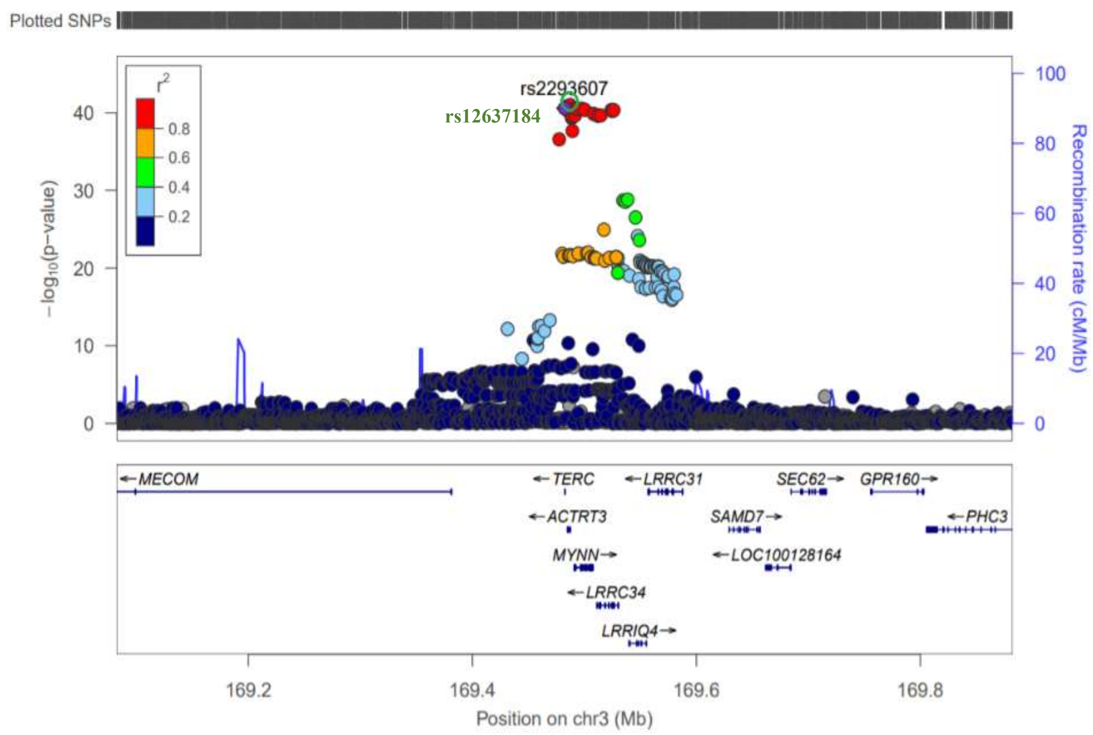

1. Dorajoo, R., X. Chang, R.L. Gurung, et al., *Loci for human leukocyte telomere length in the Singaporean Chinese population and trans-ethnic genetic studies.* Nature communications, 2019. **10**(1): p. 2491 DOI: 10.1038/s41467-019-10443-2.

Supplementary Figure 4. Regional common SNP associations at loci identified new common SNPs in linkage disequilibrium with previous reported index SNPs [1]. **a.** *PARP1* (rs3219104, chr1) **b.** *TERC* (rs2293607, chr3) **c.** *NAF1* (rs10857352, chr4) **d.** *TERF1* (rs28365964, chr8) **e.** *OBFC1* (rs12415148, chr10) and **f.** *ATM* (rs227080, chr11). Plots plotted using LocusZoom (http://csg.sph.umich.edu/locuszoom/). Previous index SNPs were indicated as purple diamond. New index SNPs were circled and indicated in green.

c

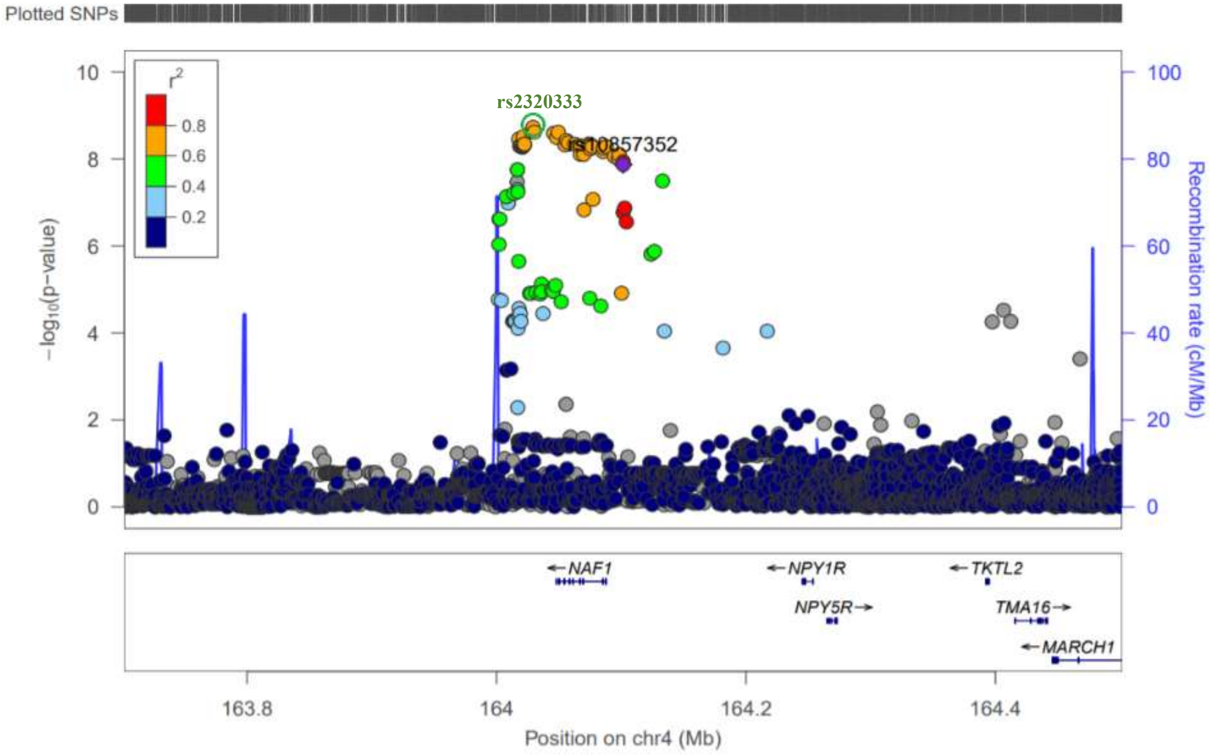

1. Dorajoo, R., X. Chang, R.L. Gurung, et al., *Loci for human leukocyte telomere length in the Singaporean Chinese population and trans-ethnic genetic studies.* Nature communications, 2019. **10**(1): p. 2491 DOI: 10.1038/s41467-019-10443-2.

Supplementary Figure 4. Regional common SNP associations at loci identified new common SNPs in linkage disequilibrium with previous reported index SNPs [1]. **a.** *PARP1* (rs3219104, chr1) **b.** *TERC* (rs2293607, chr3) **c.** *NAF1* (rs10857352, chr4) **d.** *TERF1* (rs28365964, chr8) **e.** *OBFC1* (rs12415148, chr10) and **f.** *ATM* (rs227080, chr11). Plots plotted using LocusZoom (http://csg.sph.umich.edu/locuszoom/). Previous index SNPs were indicated as purple diamond. New index SNPs were circled and indicated in green.

d

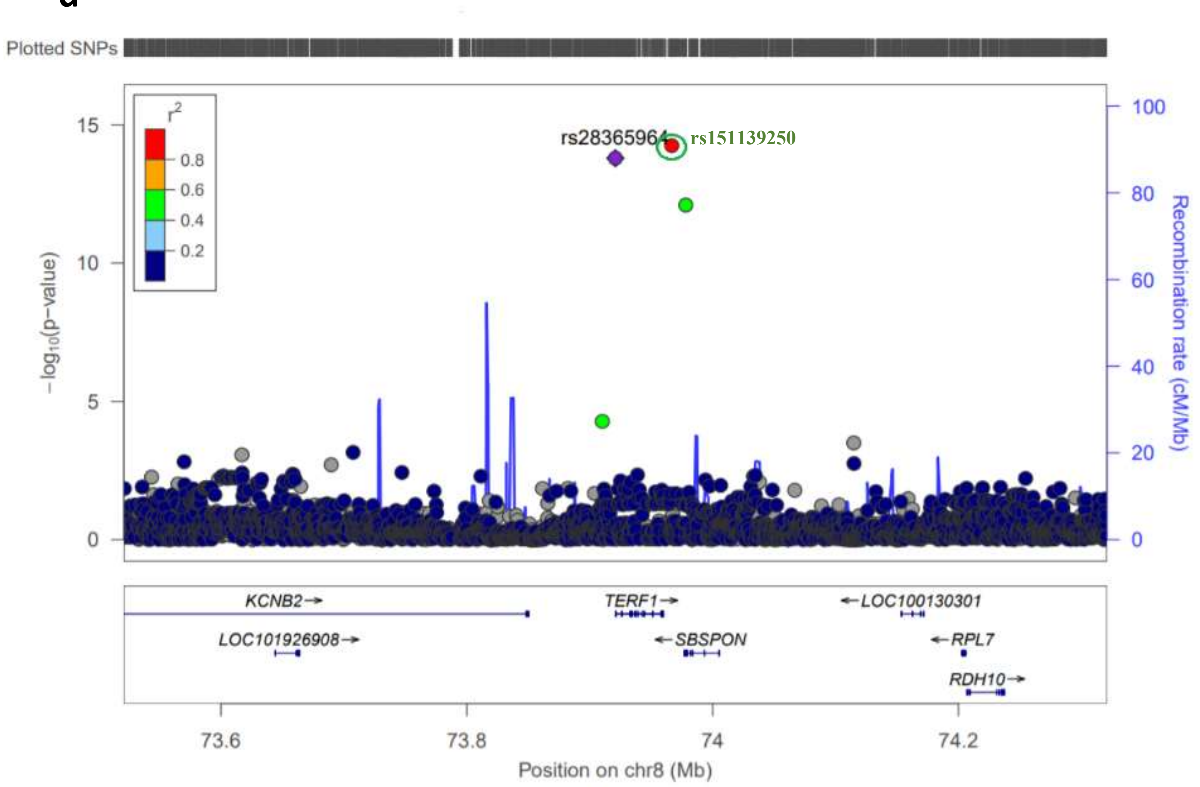

1. Dorajoo, R., X. Chang, R.L. Gurung, et al., *Loci for human leukocyte telomere length in the Singaporean Chinese population and trans-ethnic genetic studies.* Nature communications, 2019. **10**(1): p. 2491 DOI: 10.1038/s41467-019-10443-2.

Supplementary Figure 4. Regional common SNP associations at loci identified new common SNPs in linkage disequilibrium with previous reported index SNPs [1]. **a.** *PARP1* (rs3219104, chr1) **b.** *TERC* (rs2293607, chr3) **c.** *NAF1* (rs10857352, chr4) **d.** *TERF1* (rs28365964, chr8) **e.** *OBFC1* (rs12415148, chr10) and **f.** *ATM* (rs227080, chr11). Plots plotted using LocusZoom (http://csg.sph.umich.edu/locuszoom/). Previous index SNPs were indicated as purple diamond. New index SNPs were circled and indicated in green.

e

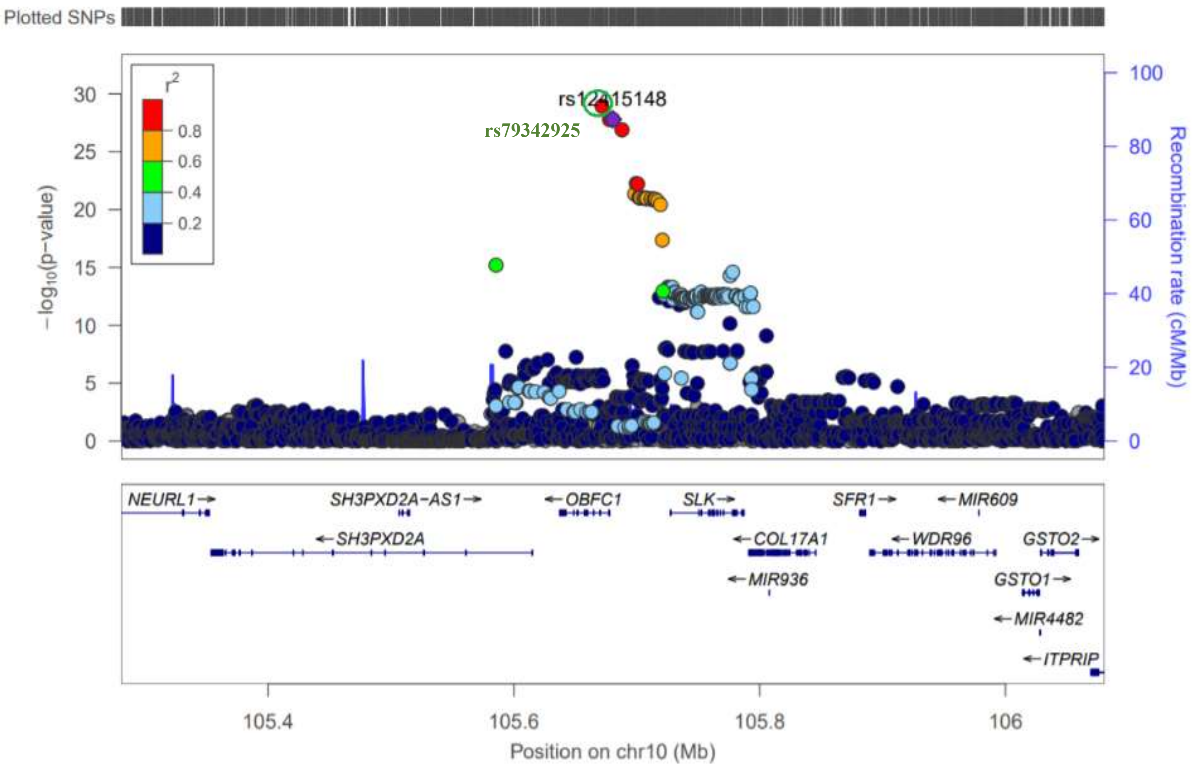

1. Dorajoo, R., X. Chang, R.L. Gurung, et al., *Loci for human leukocyte telomere length in the Singaporean Chinese population and trans-ethnic genetic studies.* Nature communications, 2019. **10**(1): p. 2491 DOI: 10.1038/s41467-019-10443-2.

Supplementary Figure 4. Regional common SNP associations at loci identified new common SNPs in linkage disequilibrium with previous reported index SNPs [1]. **a.** *PARP1* (rs3219104, chr1) **b.** *TERC* (rs2293607, chr3) **c.** *NAF1* (rs10857352, chr4) **d.** *TERF1* (rs28365964, chr8) **e.** *OBFC1* (rs12415148, chr10) and **f.** *ATM* (rs227080, chr11). Plots plotted using LocusZoom (http://csg.sph.umich.edu/locuszoom/). Previous index SNPs were indicated as purple diamond. New index SNPs were circled and indicated in green.

f

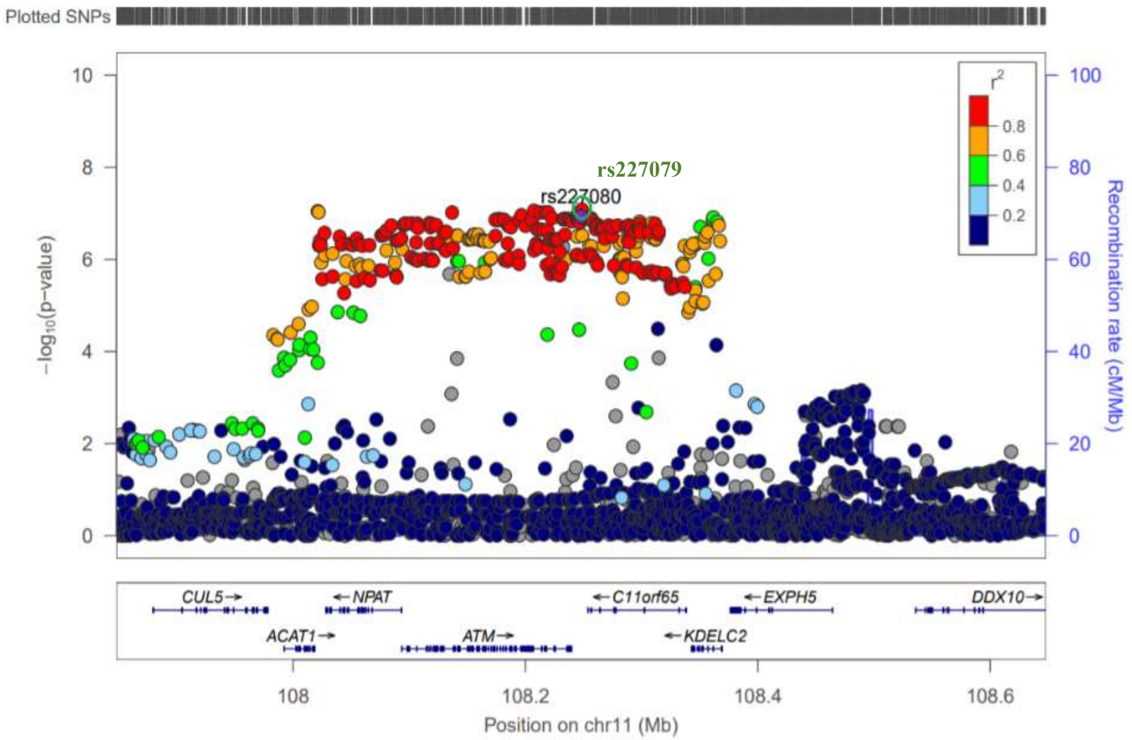

1. Dorajoo, R., X. Chang, R.L. Gurung, et al., *Loci for human leukocyte telomere length in the Singaporean Chinese population and trans-ethnic genetic studies.* Nature communications, 2019. **10**(1): p. 2491 DOI: 10.1038/s41467-019-10443-2.

Supplementary Figure 5. Regional SNP common associations at the *POT1* gene locus. **a.** Previous reported index SNP rs77767444 **b.** New index SNP rs34398311 **c.** rs34398311 condition on rs77767444. Plots plotted using LocusZoom (http://csg.sph.umich.edu/locuszoom/).

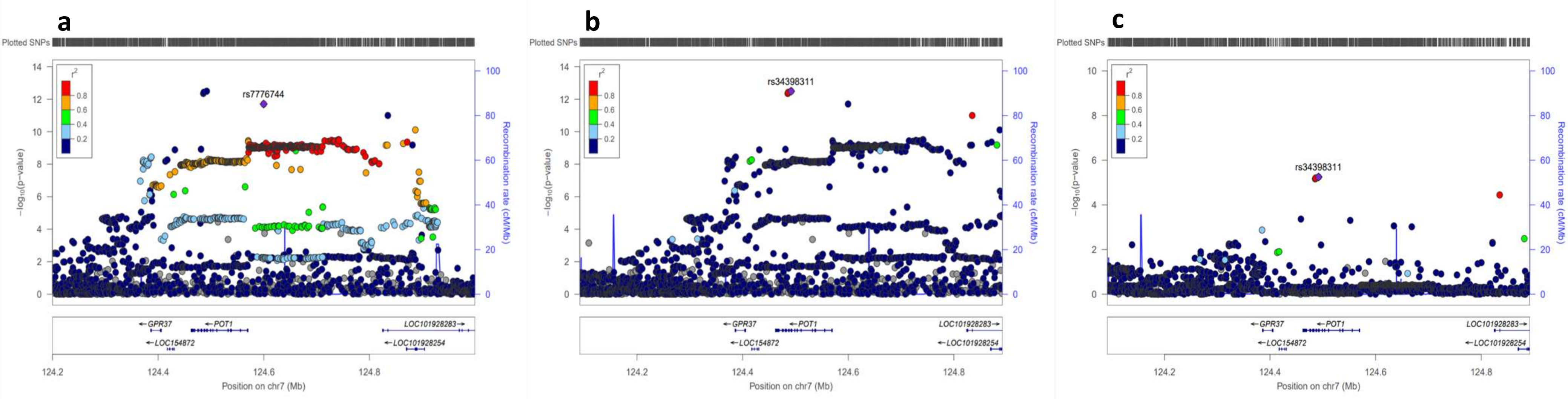

Supplementary Figure 6. Regional low frequency SNP common associations at the *POT1* gene locus. **a.** rs79314063 (genotyped missense variant, p.ASP410His) **b.** rs186524197 included in the association between LTL and rs79314063 as covariate. c**.** rs186524197 (imputed intergenic SNP) **d.** rs79314063 included in the association between LTL and rs186524197 as covariate. Plots plotted using LocusZoom (http://csg.sph.umich.edu/locuszoom/).

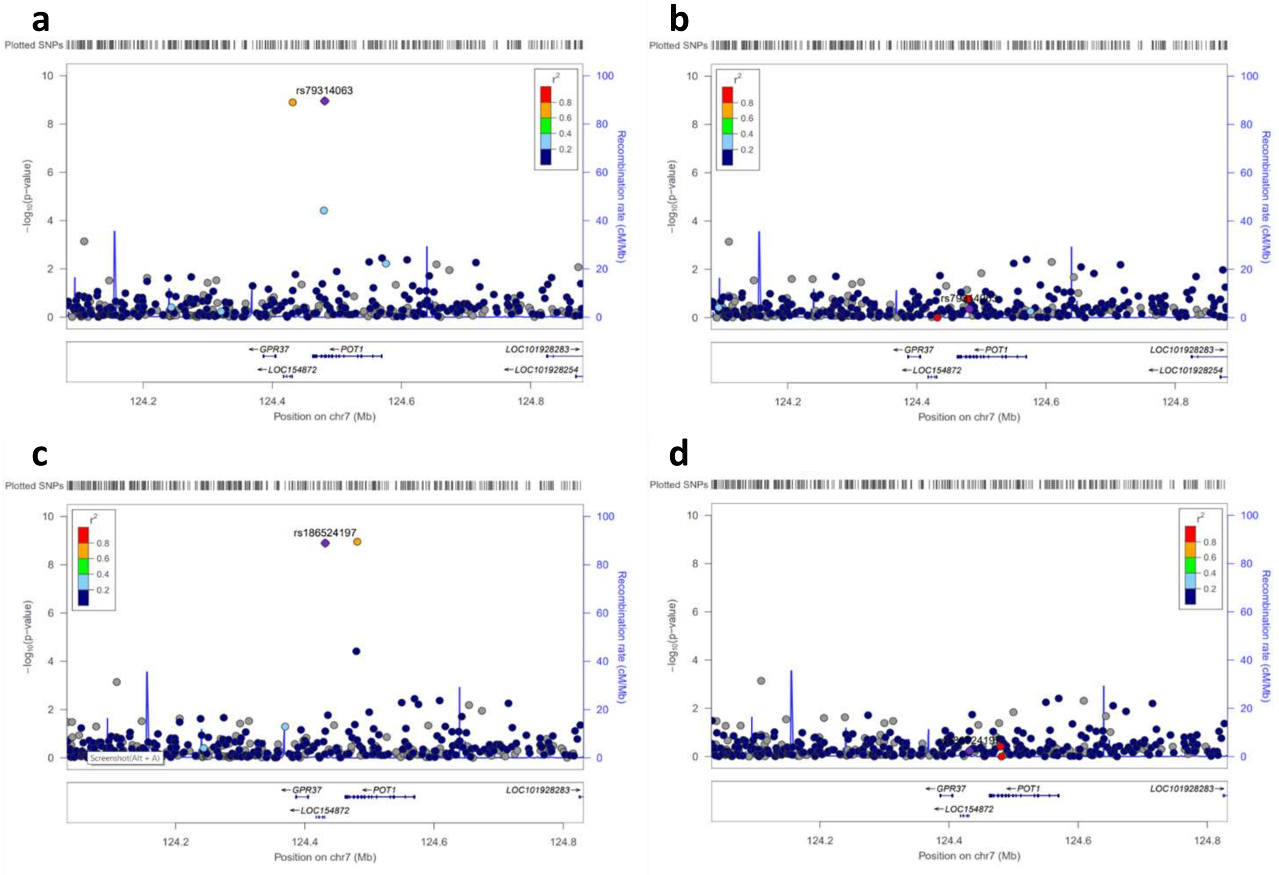

Supplementary Figure 7. 3D chromatin interaction mapping for rs79617270 (chromosome 8). **a.** Overall circos plot indicating 3 interactions at chromosome 8 with region containing rs79617270. **b.** Region 8:73960001-74000000 (indicated in red) contained rs79617270 and interacted with promoter regions of *TERF1* and *SBSPON* in blood lymphoblast cell line (GM12878, indicated in green). Diagrams plotted using FUMAGWAS (https://fuma.ctglab.nl/). Mapped genes were those where chromatin interaction loops overlap gene promoter regions (250 bp up- and 500 bp down-stream of TSS by default).

a

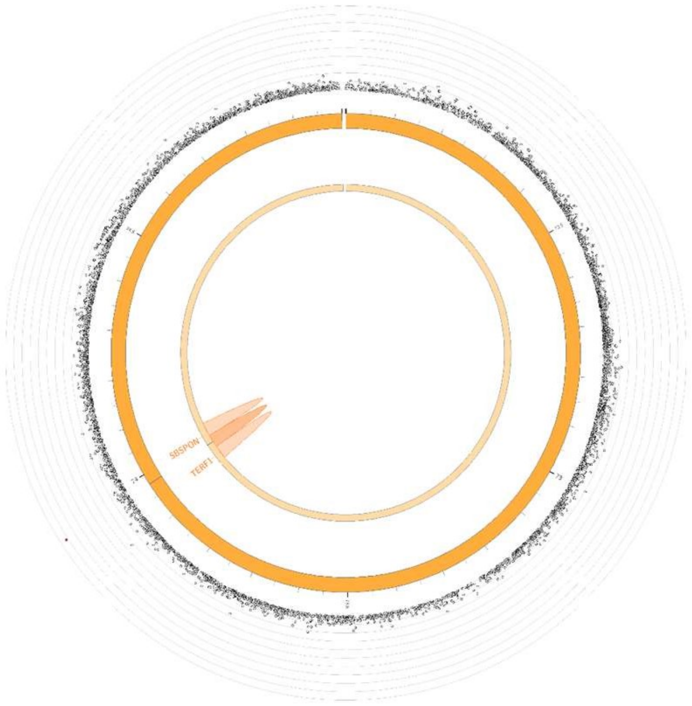

b

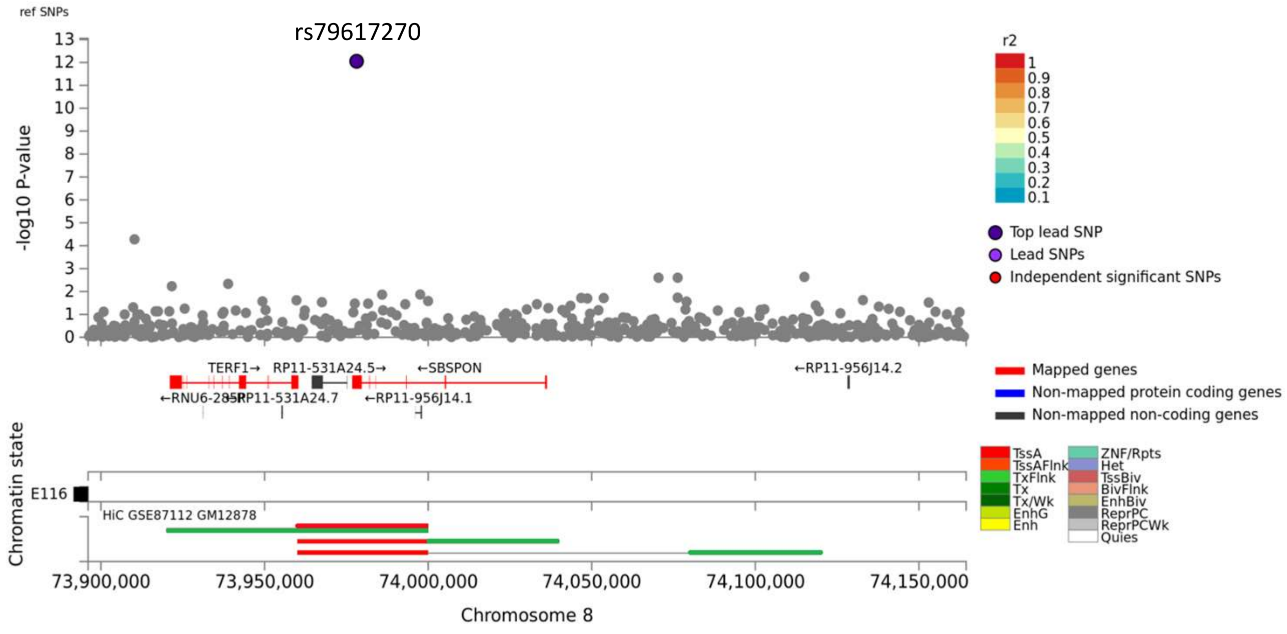

Supplementary Figure 8. 3D Chromatin Interaction mapping for rs139620151 (chromosome 10). **a.** Overall circos plot indicating 4 interactions at chromosome 10 with region containing rs139620151. **b.** Region 10:105560001-105600000 (indicated in green) contained rs139620151 and interacted with promoter elements of the proximal *STN1* (*OBFC1*) and *GSTO2* genes in lung fibroblast cells (IMR90, indicated in red). Diagrams plotted using FUMAGWAS (https://fuma.ctglab.nl/). Mapped genes were those where chromatin interaction loops overlap gene promoter regions (250 bp up- and 500 bp down-stream of TSS by default).

a

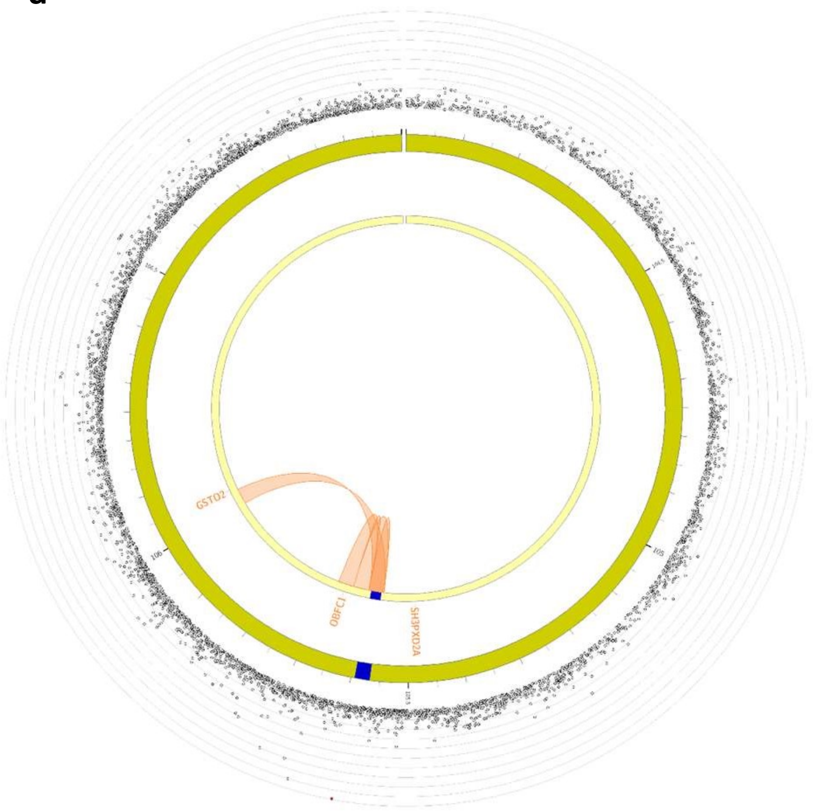

b

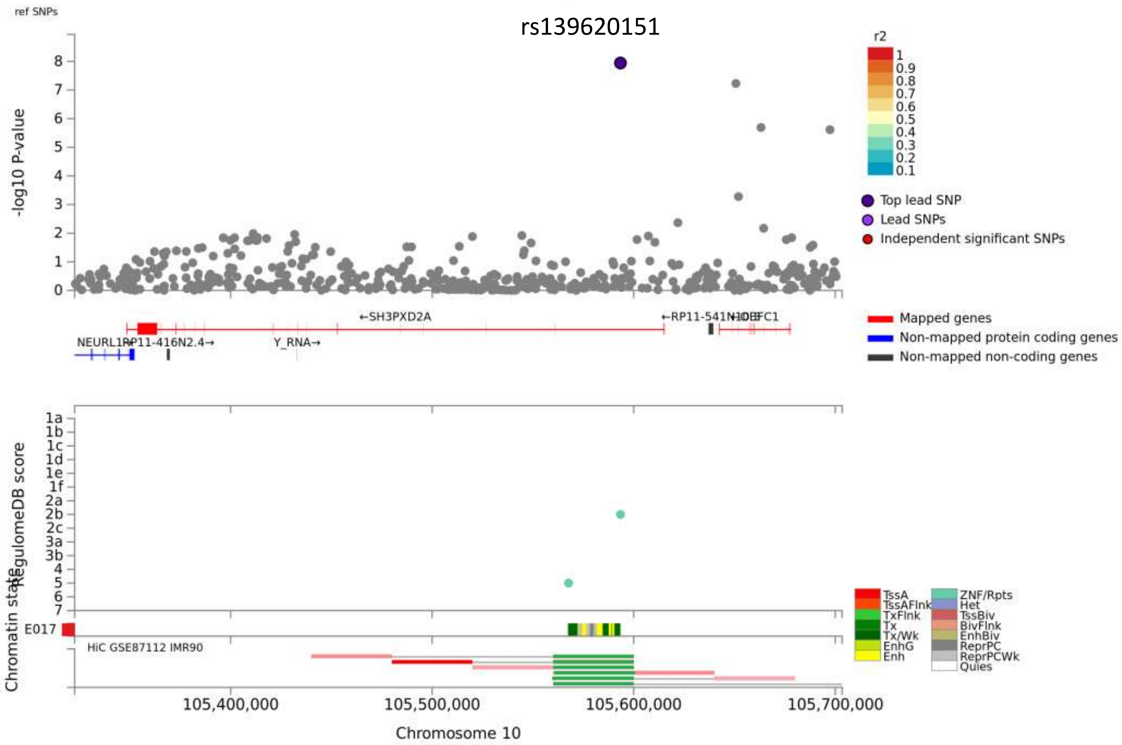

Supplement: Supplementary file 1 — Supplementary Information [file 42003_2021_2056_MOESM1_ESM.pdf]
