# Supplementary material for: Low frequency variants associated with leukocyte telomere length in the Singapore Chinese population
Source: Commun Biol. 2021 May 3;4:519. doi: 10.1038/s42003-021-02056-7 (PMC8093266; doi:10.1038/s42003-021-02056-7)
Supplement: Supplementary file 2 — Reporting Summary [file 42003_2021_2056_MOESM2_ESM.pdf]

nature research

Corresponding author(s): Rajkumar Dorajoo; Heng Chew-Kiat

Last updated by author(s): 04/03/2021

Reporting Summary

Nature Research wishes to improve the reproducibility of the work that we publish. This form provides structure for consistency and transparency in reporting. For further information on Nature Research policies, see our Editorial Policies and the Editorial Policy Checklist.

Statistics

For all statistical analyses, confirm that the following items are present in the figure legend, table legend, main text, or Methods section.

n/a	Confirmed	
☐	☒	The exact sample size (n) for each experimental group/condition, given as a discrete number and unit of measurement
☒	☐	A statement on whether measurements were taken from distinct samples or whether the same sample was measured repeatedly
☐	☒	The statistical test(s) used AND whether they are one- or two-sided<br>*Only common tests should be described solely by name; describe more complex techniques in the Methods section.*
☐	☒	A description of all covariates tested
☐	☒	A description of any assumptions or corrections, such as tests of normality and adjustment for multiple comparisons
☐	☒	A full description of the statistical parameters including central tendency (e.g. means) or other basic estimates (e.g. regression coefficient) AND variation (e.g. standard deviation) or associated estimates of uncertainty (e.g. confidence intervals)
☒	☐	For null hypothesis testing, the test statistic (e.g. F, t, r) with confidence intervals, effect sizes, degrees of freedom and P value noted<br>*Give P values as exact values whenever suitable.*
☒	☐	For Bayesian analysis, information on the choice of priors and Markov chain Monte Carlo settings
☒	☐	For hierarchical and complex designs, identification of the appropriate level for tests and full reporting of outcomes
☒	☐	Estimates of effect sizes (e.g. Cohen's d, Pearson's r), indicating how they were calculated

Our web collection on statistics for biologists contains articles on many of the points above.

Software and code

Policy information about availability of computer code

Data collection	No custom software used.
Data analysis	RVTESTS version:20150104, ANNOVAR ver201910244, R, STATA (version15), FUMA v1.3.6, IMPUTE v2 was used for data analysis.

For manuscripts utilizing custom algorithms or software that are central to the research but not yet described in published literature, software must be made available to editors and reviewers. We strongly encourage code deposition in a community repository (e.g. GitHub). See the Nature Research guidelines for submitting code & software for further information.

Data

Policy information about availability of data

All manuscripts must include a data availability statement. This statement should provide the following information, where applicable:

- Accession codes, unique identifiers, or web links for publicly available datasets
- A list of figures that have associated raw data
- A description of any restrictions on data availability

All 4,598,599 variants tested for Singapore Chinese LTL rare variants GWAS in the SCHS Discovery is available in https://figshare.com/articles/dataset/telomere_rare_variants_SCHS_txt/12951689.

Field-specific reporting

Life sciences study design

All studies must disclose on these points even when the disclosure is negative.

Sample size	We utilized all the samples with genotype and telomere measurements from the study population.
Data exclusions	No data was excluded from the analysis.
Replication	We replicated our findings in several independent datasets of the same ethnicity to valid the findings.
Randomization	Our study is a population genetics study therefore randomization is not relevant.
Blinding	Our study is a population genetics study therefore blinding is not relevant.

Reporting for specific materials, systems and methods

We require information from authors about some types of materials, experimental systems and methods used in many studies. Here, indicate whether each material, system or method listed is relevant to your study. If you are not sure if a list item applies to your research, read the appropriate section before selecting a response.

Materials & experimental systems

n/a	Involved in the study	
☒	☐	Antibodies
☒	☐	Eukaryotic cell lines
☒	☐	Palaeontology and archaeology
☒	☐	Animals and other organisms
☐	☒	Human research participants
☒	☐	Clinical data
☒	☐	Dual use research of concern

Methods

n/a	Involved in the study	
☒	☐	ChIP-seq
☒	☐	Flow cytometry
☒	☐	MRI-based neuroimaging

Human research participants

Policy information about studies involving human research participants

Population characteristics	Written informed consent was obtained from all study participants. The Institutional Review Board at the National University of Singapore and the University of Minnesota, the SingHealth Centralized Institutional Review Board and Singapore National Health Group domain-specific ethical committee. In SCHS, 27,308 SCHS samples were genotyped on the Illumina Global Screening Array (GSA). Additional replication data were drawn from 1,928 independent subjects ("SCHS CAD") from the SCHS-CAD nested case-control study with relevant LTL data genotyped on Zhonghua8Beadchip. Genome-wide genotyping was performed for DN cohort using the Illumina HumanOmniZhonghua Bead Chip. Genome-wide genotyping was performed for SMART2D using the Illumina humanOmniExpress-24 Bead Chip. For the SingHEART/Biobank study, whole genome sequencing (WGS) was performed for 154 Chinese participants.
Recruitment	In SCHS, 63,257 Singaporean Chinese participants consisting of 27,959 men and 35,298 women that were of the two major Chinese dialect groups in Singapore (the Hokkien and the Cantonese) were recruited between April 1993 and December 1998. The Singapore Study of Macro-angiopathy and Micro-vascular Reactivity in Type 2 Diabetes (SMART2D) dataset is a cross-sectional study conducted between August 2011 and February 2014, including 2,057 adults aged 21-90 years with type 2 diabetes mellitus (T2DM). The SingHEART/Biobank study was established at the National Heart Centre Singapore and is a cohort of normal volunteers enrolled to characterize normal reference values for various cardiovascular and metabolic disease-related markers in Singaporeans.
Ethics oversight	Written informed consent was obtained from all study participants. The Institutional Review Board at the National University of Singapore and the University of Minnesota, the SingHealth Centralized Institutional Review Board and Singapore National Health Group domain-specific ethical committee.

Note that full information on the approval of the study protocol must also be provided in the manuscript.
